# Arts engagement and self‐esteem in children: results from a propensity score matching analysis

**DOI:** 10.1111/nyas.14056

**Published:** 2019-04-15

**Authors:** Hei Wan Mak, Daisy Fancourt

**Affiliations:** ^1^ Department of Behavioural Science and Health University College London London UK

**Keywords:** arts engagement, children, self‐esteem, propensity score matching

## Abstract

Self‐esteem is regarded as vital to children's social and cognitive development and emotional well‐being. To date, a few studies have suggested that arts activities can improve self‐esteem in young people. However, such studies mainly focused on small, nonrepresentative samples. In this study, data from 6209 children included in the United Kingdom Millennium Cohort Study were analyzed using propensity score matching to investigate the association between children's arts engagement ((1) listening to or playing music; (2) drawing, painting, or making things; and (3) reading for enjoyment) and self‐esteem at age 11. All three activities were associated with higher levels of self‐esteem when matching for all identified demographic, socioeconomic, and familial confounders. Additionally, the relationship was more prominent when children engaged in these activities with their parents on a regular basis. However, there was no clear evidence that ability in either music or arts activities moderated the relationship with self‐esteem, although English language ability may moderate the association between reading and self‐esteem. These results suggest that initiatives to promote arts engagement in children may provide a practical and efficient way to improve children's self‐esteem. This is the key given self‐esteem in childhood tends to decline as children enter adolescence, yet is linked to lifelong development and well‐being.

## Introduction

The relationship between arts engagement and psychological well‐being and mental health is well established.^1^, ^2^, ^3^, ^4^ Among young people in particular, arts activities are associated with both mental health and well‐being^5^, ^6^, ^7^, ^8^ and also with a range of related factors, including fewer socioemotional difficulties, higher levels of academic self‐confidence, improved mood, and better communication for children with autistic spectrum disorders.^5^, ^9^, ^10^

However, a gap in the literature pertains to the relationship between arts engagement and children's self‐esteem. Self‐esteem is regarded as a key to one's social and cognitive development and emotional well‐being.^11^, ^12^, ^13^ Level of self‐esteem in the early years is associated with later self‐esteem in the future.^14^ To date, a few preliminary studies have found self‐esteem to be positively correlated with arts activities.^2^, ^3^, ^4^ But these studies have focused on short‐term art projects and involved small nonrepresentative samples. Whether regular arts engagement during childhood at a wider population level is linked with higher levels of self‐esteem remains unclear. Therefore, in this study, we explore the relationship between arts engagement, such as listening to or playing music, drawing, painting, or making things, and reading for enjoyment, and children's self‐esteem at age 11. This is a critical age, as levels of self‐esteem tend to decline over the transition from late childhood to early adolescence, partly due to social comparisons and self‐evaluation on feedback from others.^14^ So, identifying activities that support self‐esteem at this transition is pivotal.

A challenge when researching arts engagement is that this engagement is socially patterned, with girls and children from higher socioeconomic families having a higher level of engagement, and with migrant children and children with special education needs having a lower level of engagement.^15^ Demographic differences in the types of arts activities among children may lead to over‐ or underestimation of the potential effect these activities may have on children's self‐esteem. Therefore, in this study, we use a sophisticated analytical method, propensity score matching (PSM), to remove the confounding bias and to assess if there is a relationship between arts engagement and self‐esteem independent of all identified potential confounding factors.

We also explored two further research questions. First, we tested whether any relationship might be moderated by parental levels of participation in these activities with their children. The implication of parental involvement in children's arts engagement has historically received little research attention, although benefits of parents’ involvement in home learning activities (e.g., teaching songs, painting, and drawing) and children's attainment and positive social behavior have been shown,^16^ and parental support and encouragement (which could be reflected in parental engagement) in arts activities are associated with a higher level of children's enjoyment during participation that may affect their self‐esteem.^17^ Second, we explored whether children's abilities in arts activities might moderate levels of self‐esteem. The “self‐efficacy” hypothesis suggests that individuals are likely to engage in arts activity if they believe that (or are presented with information that) they are adequate or capable in doing so and that such activity is not seen as a threat to their sense of self.^18^ Given this, it has been suggested that self‐esteem could be more the result of doing well,^12^ rather than the result of arts engagement. So we also examined the relationship between ability in arts activities and children's self‐esteem among a sample who frequently engaged in these activities.

## Methods

### Participants

This study analyzed data from the Millennium Cohort Study (MCS): a longitudinal study following a nationally representative sample of 18,818 infants born in the United Kingdom who were 9 months old in 2000–2001 with a follow‐up every 2–3 years. This study focused on Sweep 5 (2011) interviews when sample members were aged 11.^19^, ^20^ However, it also drew on some data from Sweep 4 (2008) when participants were aged 7. A total of 13,469 children were included in Sweep 5 (a response rate of 81%). In our analysis, we only considered participants who were not born as twins or triplets (less than 1.5% were twins/triplets; *n* = 13,287). We also only included participants with natural parents (*n* = 12,889). To ensure a higher quality of matching for the subsequent PSM analysis (fuller information about the children and a more homogenous sample that helped reduce unobserved bias), we further restricted the sample to those for whom both their mothers and fathers had participated in the survey so that we could model the parent–child interactions and behaviors for both parents and take account of both parents’ mental health (*n* = 6686; although sensitivity analyses confirmed findings in the larger sample, too). Of these, 6209 participants provided full data across all measures so were included in analyses (Table 1). The MCS receives ethical approval from the National Research Ethics Service and all participants provide informed consent.

**Table 1:** Descriptive statistics of outcome and control variables (*n* = 6209) from MCS data

		Mean (SD) or %
Self‐esteem, Sweep 5	Self‐esteem	0.079 (0.949)
Demographic variables	Female	50.10
	Ethnicity: White	89.32
	[P] Married/remarried/in a civil partnership	90.96
	[P] Single, never married, and never in a civil partnership^2^	6.97
	[P] Legally separated/divorced/widowed/in a surviving civil partnership	2.06
Socioeconomic status	[P] NVQ Level 1	8.42
	[P] NVQ Level 2	29.51
	[P] NVQ Level 3	7.65
	[P] NVQ Level 4	28.41
	[P] NVQ Level 5	12.18
	[P] Overseas/none of these	13.83
	[P] Semiroutine and routine	17.09
	[P] Lower supervisory and lower technician^4^	9.78
	[P] Small employers and self‐employed	18.23
	[P] Intermediate	6.20
	[P] Managerial/professional	48.70
Relationships with parents	Closeness with mother^a^	−0.015 (0.969)
	Closeness with father^a^	0.007 (0.996)
Parents’ depression in the past 30 days	Mother feeling depressed^a^	−0.201 (0.812)
	Father feeling depressed^a^	−0.069 (0.910)
Parental engagement in arts and cultural activities with children, Sweep 4	Do musical activities with children^b^	4.806 (1.230)
	Paint, draw, or make things with children^b^	3.722 (1.074)
	Read to children^b^	5.268 (0.925)
Strengths and difficulties, Sweep 4	Prosocial behavior	0.090 (0.920)
	Emotional problems	−0.126 (0.895)
	Peer problems	−0.140 (0.857)
	Conduct problems	−0.186 (0.837)
	Hyperactivity/inattention	−0.164 (0.911)
Reason for choosing school, Sweep 5	School offers a good range of extracurricular activities	31.08

Notes: The table shows means and standard deviations in parentheses or percentages.

[P] represents parent.

a Higher scales indicate a greater level of closeness/feeling depressed.

b A 6‐point scale, ranging from “not at all” to “every day or almost every day.”

### Measures

Children's self‐esteem was measured in Sweep 5 via bespoke survey instruments which draw on Rosenberg's 10‐item self‐esteem scale.^21^ The measurement is based on five items, all self‐reported by children (α= 0.83), and standardized to have a mean 0 and a standard deviation of 1.

Arts engagement was measured in Sweep 5 through asking children how often they (1) listen to or play music, (2) draw, paint, or make things, and (3) read for enjoyment, not for school but as an extracurricular activity at home (Table 2). Responses were measured on a 5‐point scale, ranging from “never” to “most days.” Our main model was based on a binary indicator for each activity of whether children were engaged in these activities most days. We also generated another set of binary indicators focused on extremes of engagement (1 = doing the activity most days, while 0 = doing the activity never or less often than once a month). To minimize the hidden bias that might be found during the interval between the interviews,^22^ the outcome and the independent variables were derived from the same sweep.

**Table 2:** Relationship between arts and cultural engagement, outside school, and self‐esteem in Sweep 5 (age 11)

		Listen to/play music	Paint, draw, or make things	Read for enjoyment
Most days versus otherwise	ATT	0.072 (0.023)*	0.139 (0.029)**	0.136 (0.025)**
	Mean bias (%)	0.6	0.6	0.6
	Rubin's B	4.3	3.9	4.7
	Rubin's R	1.14	1.18	1.16
	Treatment, *n*	3673	1405	3079
	Control, *n*	2535	4804	3121
	Total, *n*	6208	6209	6200
Most days versus never/less often than once a month	ATT	0.162 (0.052)*	0.319 (0.043)**	0.224 (0.059)**
	Mean bias (%)	3.1	2.7	2.3
	Rubin's B	18.6	14.1	16.8
	Rubin's R	1.27	1.03	1.50
	Treatment, *n*	3642	1382	3077
	Control, *n*	502	1289	751
	Total, *n*	4144	2671	3828

Notes: Columns present ATT estimates from PSM models using Epanechnikov kernel matching with 0.05 bandwidths; common support condition was imposed. The models controlled all covariates. ATT standard errors in parentheses were computed by bootstrapping with 100 replications.

Statistical significance is denoted by asterisks: ^*^, significant at 1%; ^**^, significant at 0.1%.

Success of the PSM was assessed using Rubin's B <25%, Rubin's R of 0.5−2, and a percentage bias of <10% for each covariate.

Children's ability in music, arts, and design, and English language was rated by their teachers, with binary variables for each, respectively, denoting children who performed the activity above average/well above average versus children who performed average or below.

We used PSM to match our sample on all identified factors that could confound the relationship between children's arts engagement and their self‐esteem, including (1) children's gender and ethnicity; parental marital status, educational level, and employment status; (2) parents’ self‐rated closeness of relationship with child (ranging from “not very close” to “extremely/very close”; standardized); (3) mothers’ and fathers’ mental health (K6 Questionnaire; standardized);^23^ and (4) parental perception regarding the school offering a good range of extracurricular activities (e.g., music, dancing, and acting) as an important factor in choosing a secondary school.^15^ All factors were measured in Sweep 5. We further included (5) children's Strengths and Difficulties Questionnaire (SDQ) measured in Sweep 4 in order to control for previous behaviors that could affect both reading and self‐esteem. This included scales assessing prosocial behavior, emotional problems, peer problems, conduct problems, and hyperactivity/inattention (five scales derived from 25 items; all standardized).^24^ (6) Parental engagement in arts activities with children was also considered (measured using a 6‐point measure, ranging from “not at all” to “every day or almost every day”); the variable was only measured in Sweep 4. However, this was, in fact, supportive for the analyses as we expect strong correlations between parents’ and children's engagement in arts. So, if parental engagement was measured at the same time as the independent variables, we might encounter multicollinearity issues, which could lead to biased estimations.

### Statistics

Our analysis used PSM. PSM addresses the previous analytical issue of traditional regression models that certain types of children may be disproportionately more likely to be in the “frequent arts engagement” group by estimating the propensity of each child to be in this group based on the factors (e.g., gender and socioeconomic backgrounds) that may determine children's involvement in these activities. Propensity scores are then used to pair each child who engages in arts activities most days (designated as the “treatment” group) with one or more children who engage in these activities less often (designated as the “control” group) and who have almost identical distributions on all observed covariates. This means that those covariates that may confound the association between children's engagement and self‐esteem would no longer be influential. The average difference in self‐esteem score between the treatment (children who engaged in arts most days) and the control (children who engaged in arts less often than most days) groups can be interpreted as the difference attributed to children's frequency of participation in arts activities. PSM provides an estimate of the average treatment effect on the treated (ATT), which is the difference between the average outcome for children who participate in arts activities most days, and the average outcome for the same group under the hypothetical scenario that they participated less often. In this way, PSM simulates a randomized trial for situations, where randomized trials are infeasible or unethical.^22^

Our primary analyses explored the relationship between arts engagement and self‐esteem among children with different frequencies of involvement. We carried out two secondary analyses. First, we examined whether the relationship differed depending on whether parents were engaged in these activities with their children. To do this, for each parent−child activity, we generated a binary variable in order to divide the sample into two groups: high levels of parental involvement and low levels of parental involvement (Tables 3 and 4). Second, we explored whether, among those children who engaged most days, their abilities in the arts activities had a differential relationship with their self‐esteem. To do this, we restricted the sample just to those children who engaged with arts activities most days and created a new propensity match based on whether the children had above average/well above average versus average/below average abilities in those activities as rated by their teachers (Table 5).

**Table 3:** Classifications of high and low levels of parental engagement in arts and cultural activities with children in Sweep 4 (age 7)

	Listen to/play music	Paint, draw, or make things	Read for enjoyment
High parental engagement	At least once or twice a week or more	At least once or twice a week or more	Every day or almost every day
Low parental engagement	Once or twice a month or less	Once or twice a month or less	Once or twice a week or less

**Table 4:** Relationship between arts and cultural engagement, outside school, in Sweep 5 and self‐esteem (age 11): parental engagement in arts and cultural activities with children in Sweep 4 (age 7)

		Listen to/play music	Paint, draw, or make things	Read for enjoyment
Most days versus otherwise	High parental engagement in Sweep 4			
	ATT	0.068 (0.025)*	0.155 (0.036)**	0.141 (0.034)**
	Mean bias (%)	1.0	0.6	1.0
	Rubin's B	6.3	4.4	7.3
	Rubin's R	1.15	1.15	1.17
	Treatment, *n*	3255	893	1749
	Control, *n*	2122	2563	1471
	Total, *n*	5377	3456	3220
	Low parental engagement in Sweep 4			
	ATT	0.079 (0.060)	0.117 (0.045)*	0.062 (0.069)
	Mean bias (%)	1.7	1.1	1.5
	Rubin's B	10.1	7.0	10.1
	Rubin's R	0.95	1.23	1.05
	Treatment, *n*	412	512	505
	Control, *n*	413	2241	719
	Total, *n*	825	2753	1224
Most days versus, never/less often than once a month	High parental engagement in Sweep 4			
	ATT	0.175 (0.064)*	0.381 (0.057)**	0.320 (0.064)**
	Mean bias (%)	2.9	2.9	3.7
	Rubin's B	19.8	18.2	21.0
	Rubin's R	1.38	1.16	1.21
	Treatment, *n*	3236	873	1639
	Control, *n*	383	631	318
	Total, *n*	3619	1504	1957
	Low parental engagement in Sweep 4			
	ATT	0.106 (0.110)	0.240 (0.066)**	0.162 (0.098)
	Mean bias (%)	4.9	2.0	4.0
	Rubin's B	32.9	12.9	27.8
	Rubin's R	0.97	1.10	1.26
	Treatment, *n*	406	502	488
	Control, *n*	119	658	222
	Total, *n*	535	1160	710

Notes: Columns present ATT estimates from PSM models using Epanechnikov kernel matching with 0.05 bandwidths; common support condition was imposed. The models controlled all covariates. ATT standard errors in parentheses were computed by bootstrapping with 100 replications.

Statistical significance is denoted by asterisks: ^*^, significant at 1%; ^**^, significant at 0.1%.

Success of the PSM was assessed using Rubin's B <25%, Rubin's R of 0.5−2, and a percentage bias of <10% for each covariate.

**Table 5:** Relationship between ability rated by teacher (above average/well above average versus average or below) and self‐esteem in Sweep 5 among children who engage in arts and cultural activities most days (age 11)

Arts and cultural activities	Listen to/play music	Paint, draw, or make things	Read for enjoyment
Ability rated by teacher	Music	Arts and design	English language
ATT	0.100 (0.056)	−0.056 (0.077)	0.122 (0.056)†
Mean bias (%)	0.9	1.7	1.9
Rubin's B	6.6	12.3	12.9
Rubin's R	1.22	0.87	1.08
Treatment, *n*	651	326	1190
Control, *n*	1443	462	616
Total, *n*	2094	788	1866

Notes: Columns present ATT estimates from PSM models using Epanechnikov kernel matching with 0.05 bandwidths; common support condition was imposed. The models controlled all covariates. ATT standard errors in parentheses were computed by bootstrapping with 100 replications.

Statistical significance: ^†^, significant at 5%.

Success of the propensity score matching was assessed using Rubin's B <25%, Rubin's R of 0.5–2, and a percentage bias of <10% for each covariate.

PSM models were implemented using Stata *psmtach2* and *pstest* commands. We used kernel matching method with 0.05 bandwidths; an approach that uses all available observations and constructs a weighted average of counterfactuals for each observation in the treatment group.^22^ This means that more information was taken from the matches whose propensity scores were closer to each other and less information from those whose propensity scores were distal from each other.^22^ The common support condition was imposed to remove bad quality of matching pairs; this further ensured the quality of the matches.^25^ Standard errors for the ATT were computed using bootstrapping with 100 repetitions to obtain more conservative standard errors.

Results for all PSM models demonstrated high standard matching (Morgan^26^ and Rubin^27^ define quality of matching as a value < 25% for Rubin's B, a value between 0.5 and 2 for Rubin's R, and a percentage bias <10% for each covariate), indicating that the ATT estimates obtained from the PSM models were efficient and that the observed bias was minimal. Overall, *P* values < 0.01 were considered significant.

As a sensitivity analysis, we reran analyses not restricting the sample to children with data available from both mothers and fathers but including those without data from their father. This increased the sample size available for matching to 9109. However, it meant that we could not match on fathers’ mental health or relationship with their children. Results from these analyses are included as Supplementary Material (online only).

## Results

### Demographic backgrounds

Table 1 reports the descriptive statistics of outcome and control variables. In our sample, 50% of the children were female, 89% of them were of White ethnicity, and 54% had parents with intermediate or managerial/professional employment. On average, parents tended to engage with their children in arts activities once or twice a month or more, and 31% of the parents reported that a good range of extracurricular activities offered by a school was an important factor for school selection. Before matching, there was a significant difference across all the covariates between children who participated in arts activities most days compared to those who participated less often. However, these differences were removed after matching and that the only observed difference was the frequency of participation.

### Is arts engagement associated with self‐esteem?

Table 2 represents results from the PSM models. Among the matched sample, children who participated in arts activities most days were significantly more likely to have higher levels of self‐esteem than those who participated less often (listen to/play music: ATT = 0.07, *SE* = 0.02, *P* < 0.01; paint, draw, and make things: ATT = 0.14, *SE* = 0.03, *P* < 0.001; read for enjoyment: ATT = 0.14, *SE* = 0.03, *P* < 0.001). The size of ATT was doubled when the treatment and control groups were more distinct, in which children who engaged in these activities most days were compared with those who never engaged or engaged less often than once a month (listen to/play music: ATT = 0.16, *SE* = 0.05, *P* < 0.01; paint, draw, and make things: ATT = 0.32, *SE* = 0.04, *P* < 0.001; read for enjoyment: ATT = 0.22, *SE* = 0.06, *P* < 0.001).

### Does parental engagement in these activities with children moderate the relationship between arts engagement and self‐esteem?

Table 4 presents results from the models after the sample is being split into two groups: high levels of parental engagement in arts activities and low levels of parental engagement (see Table 3 for definition). Again, we performed the analysis twice using different specifications for the control group (most days versus less than most days in panel 1; most days versus never/less often than once a month in panel 2). The relationship between children's arts activities and self‐esteem varied between the levels of parental engagement in these activities. Among children whose parents were involved in arts activities with them at least once or twice a week, their frequent engagement in these activities was positively and significantly associated with their levels of self‐esteem (listen to/play music: ATT = 0.07, *SE* = 0.03, *P* < 0.01; paint, draw, and make things: ATT = 0.16, *SE* = 0.04, *P* < 0.001; read for enjoyment: ATT = 0.14, *SE* = 0.03, *P* < 0.001). In line with our main analysis, the ATT estimates were larger between groups that have distinct levels of frequency in the activities (listen to/play music: ATT = 0.18, *SE* = 0.06, *P* < 0.01; paint, draw, and make things: ATT = 0.38, *SE* = 0.06, *P* < 0.001; read for enjoyment: ATT = 0.32, *SE* = 0.06, *P* < 0.001). However, the relationship between children's arts engagement and self‐esteem was attenuated when parents engaged less often (*Most days versus otherwise*: listen to/play music: ATT = 0.08, *SE* = 0.06, *P* > 0.05; paint, draw, or make things: ATT = 0.12, *SE* = 0.05, *P* < 0.01; read for enjoyment: ATT = 0.06, *SE* = 0.07, *P* > 0.05. *Most days versus never/less often than once a month*: listen to/play music: ATT = 0.11, *SE* = 0.11, *P* > 0.05; paint, draw, or make things: ATT = 0.24, *SE* = 0.07, *P* < 0.001; read for enjoyment: ATT = 0.16, *SE* = 0.10, *P* > 0.05). The only activity for which the relationship between child engagement and self‐esteem was preserved irrespective of parental engagement was painting, drawing, or making things.

### Among children with high levels of engagement, does ability moderate the relationship between arts activities and self‐esteem among children?

The final analysis in our study addressed the implicit assumption that children's self‐esteem might be influenced by their levels of ability in the activity (Table 5). Of children who were engaged in arts activities most days, no association was found between their ability in music or arts and design and their levels of self‐esteem. Of children who read most days, there was an indication that their English language ability was positively related to their self‐esteem (ATT = 0.12, *SE* = 0.06, *P* < 0.05).

### Sensitivity analysis

Our first sensitivity analysis removed the matching variables for fathers, which increased our overall sample size. These results entirely replicated the results from the analyses for our first and second research questions (see Tables S1 and S2, online only). For our third research question (for the role of the ability, see Table S3, online only), when not matching on fathers’ mental health or relationship with their children, the results for reading and art were replicated, but there was an indication that ability could moderate the relationship between listening to/playing music and self‐esteem (ATT = 0.111, *SE* = 0.043, *P* < 0.05).

## Discussion

Prior research has shown that arts participation is associated with self‐esteem,^1^, ^2^, ^3^, ^4^ although many of the studies tend to focus on small and targeted samples across short‐term art interventions. This paper shows that, after matching children who do and do not engage regularly on all identified confounding variables in order to remove the possibility of self‐selection into arts activities, listening to/playing music, drawing, painting, or making things, and reading most days of the week are all positively related to a higher level of self‐esteem. This relationship is more prominent when comparing children with substantially different frequencies of participation. For musical activities or reading, this relationship is only present when parents also engage in these activities with their children on a regular basis, whereas for painting, drawing, or making things, the relationship with self‐esteem is present irrespective of parental engagement. Further, there is little evidence that ability in either music or arts activities affects self‐esteem, but English language ability may modulate the relationship between reading and self‐esteem.

Overall, this research is supported by previous theoretical work on why arts activities could support self‐esteem in young people, with two broad explanations: (1) self‐identity and (2) social identity. In relation to self‐identity, arts activity (creating new arts, in particular) has been suggested to validate the uniqueness of an individual, which gives rise to a sense of accomplishment and to feelings of self‐worth in their own abilities and helps enhance self‐empowerment, self‐esteem, and self‐worth.^2^ Arts also allow distractions in which the individual will feel more in control of their environment and provide opportunities for ongoing challenges and skills improvement that could potentially be beneficial to one's self‐esteem.^8^, ^9^ In relation to social identity, the arts have been shown to support a sense of social identity and foster social cohesion through making arts as a group (e.g., choral singing) or using arts for communication.^2^, ^6^, ^28^, ^29^, ^30^, ^31^, ^32^ Establishing positive distinctiveness for the group (e.g., choral singing) which people belong enhances a positive social identity for in‐group members and hence elevates self‐esteem.^33^ The arts also help to improve pride, encourage goal‐directed behavior, and enhance social resilience which supports individuals to solve other social issues and establish self‐esteem.^8^

It is also notable that we found some variations in the relationship between arts participation and self‐esteem depending on the level of parental engagement in these activities. Our finding is in line with other parallel studies that show parental involvement in children's education and sport participation has a significant and positive effect on academic achievement and sport performance.^34^, ^35^, ^36^ Parental involvement in arts activities allows parents to spend time with their children, and helps facilitate trust and cooperation through mutual engagement, which are important for children's positive mental well‐being development, including self‐esteem (as also suggested by Papinczak *et al*.,^7^ who have shown a positive correlation between music playing, relationship building with family, and well‐being). Interestingly, the analysis shows that the association between children's engagement in painting, drawing, or making things and their self‐esteem does not vary greatly between the levels of parental involvement in these activities. One possible explanation for this could be that activities that involve creating new arts may help make people feel unique and thus foster one's self‐esteem, regardless or not whether the activities are engaged with parents.^2^

Our final analysis of the association between children's ability in arts and self‐esteem makes an important and useful contribution to the existing literature. Broadly, we found that it is not necessary for children to be good at arts in order for there to be a relationship with self‐esteem. Engagement, not ability, appears to be the key. When applying a more lenient threshold for significance, there was an indication that English language ability was associated with self‐esteem in reading, and when not matching on father's mental health or relationship with the child, there was also an indication that ability in music could be linked with self‐esteem. However, these results are somewhat inconclusive, so remain to be explored further. This is the first known study to have explored the relationship between ability and self‐esteem when engaging in arts activities. In particular, for art (for which there was no indication of any relationship between ability and self‐esteem), this finding is of a great policy importance as it could potentially serve as a motivation to encourage a wider population to participate in arts activities. Indeed, a report from ART31 (2018), which was commissioned by the Arts Council England, shows that fear of failure is the biggest barrier to young people engaging in the arts.^37^

Our study has several strengths. It is based on a nationally representative sample, with rich data on different aspects of respondents’ lives. We use an advanced statistical model, PSM, to remove possible heterogeneity that may be found in the relationship between arts engagement and self‐esteem. Our secondary analyses focused on more homogenous groups (individuals with similar levels of parental engagement in the arts and of similar levels of ability), which removed heterogeneity in the sample.^38^ Our findings make a novel contribution to the state of knowledge by assessing various types of arts activities and their relationships with children's self‐esteem. However, our study is not without its limitations. First, this analysis was cross‐sectional, so while we could describe the relationship between arts engagement and self‐esteem, causality cannot be assumed. We drew on existing preliminary data and theory suggesting that arts engagement affects self‐esteem rather than the reverse. However, it remains possible that self‐esteem could contribute to the frequency of engagement in the arts. So, this remains to be explored further in longitudinal studies. Our use of PSM as a statistical technique allowed us to match children who did and did not engage in order to assess if there is an independent relationship between arts engagement and self‐esteem. However, PSM is unable to control for the unobserved factors that may affect children's engagement and/or self‐esteem. That said, the richness of the data set has allowed the matching to a high standard, meaning that any remaining unobserved heterogeneity should be relatively small. Additionally, our cutting point for dividing the sample into the “treatment” and the “control groups” may be arbitrary; (the same goes to the division of the levels of parental engagement). Although we have addressed this issue partially by testing different thresholds for the control group, future studies might like to consider in more detail the frequency thresholds for arts activities. Furthermore, our study only investigates the children's self‐esteem at age 11. Further research is required to investigate if engagement with arts activities is also associated with higher self‐esteem when children proceed to adolescence.

## Conclusions

Our paper provides insights into the relationship between children's arts engagement and self‐esteem. Our findings show that arts activities have a significant association with children's self‐esteem and that children may experience higher self‐esteem if their parents are also involved in arts activities with them. We also find that the relationship between arts activities and self‐esteem is not clearly influenced by children's abilities; rather, the engagement itself offers a variety of benefits that enhance one's self‐esteem. While PSM controls for observable factors, the causality of the association cannot be absolutely determined. However, the relevance of this research to the design and delivery of arts programs for health is clear: arts engagement may well be important in supporting children's self‐esteem—a core marker of positive development—and thereby may play a role in reducing children's inequalities as they enter adulthood.

## Competing interests

The authors declare no competing interests.

## Supporting information


**Table S1**. Relationship between arts and cultural engagement, outside school, and self‐esteem in Sweep 5 (age 11)

**Table S2**. Relationship between arts and cultural engagement, outside school, in Sweep 5 and self‐esteem (age 11): Parental engagement in arts and cultural activities with children in Sweep 4 (age 7)

**Table S3**. Relationship between ability rated by teacher (above average/well above average versus average or below) and self‐esteem in Sweep 5 among children who engage in arts and cultural activities most days (age 11)
