# Supplementary material for: Arts engagement and self‐esteem in children: results from a propensity score matching analysis
Source: Ann N Y Acad Sci. 2019 Apr 15;1449(1):36–45. doi: 10.1111/nyas.14056 (PMC6767447; doi:10.1111/nyas.14056)
Supplement: Supplementary file 1 — Table S1. Relationship between arts and cultural engagement, outside school, and self‐esteem in Sweep 5 (age 11) [file NYAS-1449-36-s001.docx]

| **Table S1. Relationship between arts and cultural engagement, outside school, and self-esteem in Sweep 5 (age 11)** | | | | |
| --- | --- | --- | --- | --- |
|  |  | **Listen to/play music** | **Paint, draw, or make things** | **Read for enjoyment** |
| **Most days vs otherwise** | **ATT** | 0.089 (0.020)** | 0.123 (0.024)** | 0.152 (0.023)** |
|  | **Mean bias (%)** | 0.6 | 0.7 | 0.7 |
|  | **Rubin’s B** | 4.2 | 4.5 | 4.5 |
|  | **Rubin’s R** | 1.18 | 1.13 | 1.13 |
|  | **Treatment N** | 5477 | 2149 | 4310 |
|  | **Control N** | 3632 | 6960 | 4799 |
|  | **Total N** | 9109 | 9109 | 9109 |
| **Most days vs never/less often than once a month** | **ATT** | 0.200 (0.054)** | 0.285 (0.034)** | 0.252 (0.040)** |
|  | **Mean bias (%)** | 2.2 | 1.8 | 2.5 |
|  | **Rubin’s B** | 13.4 | 10.7 | 14.5 |
|  | **Rubin’s R** | 1.17 | 1.19 | 1.30 |
|  | **Treatment N** | 5477 | 2149 | 4310 |
|  | **Control N** | 746 | 1909 | 1231 |
|  | **Total N** | 6223 | 4058 | 5541 |
| Notes: Columns present ATT estimates from PSM models using Epanechnikov kernel matching with 0.05 bandwidths; common support condition is imposed. The models controlled all covariates. ATT standard errors in parentheses were computed by bootstrapping with 100 replications.  ** significance at 0.1%.  Success of the propensity score matching was assessed using Rubin’s B<25%, Rubin’s R of 0.5-2, and a percentage bias of <10% for each covariate. | | | | |
