# Supplementary material for: Arts engagement and self‐esteem in children: results from a propensity score matching analysis
Source: Ann N Y Acad Sci. 2019 Apr 15;1449(1):36–45. doi: 10.1111/nyas.14056 (PMC6767447; doi:10.1111/nyas.14056)
Supplement: Supplementary file 2 — Table S2. Relationship between arts and cultural engagement, outside school, in Sweep 5 and self‐esteem (age 11): Parental engagement in arts and cultural activities with children in Sweep 4 (age 7) [file NYAS-1449-36-s002.docx]

| **Table S2 Relationship between arts and cultural engagement, outside school, in Sweep 5 and self-esteem (age 11): Parental engagement in arts and cultural activities with children in Sweep 4 (age 7)** | | | | | |
| --- | --- | --- | --- | --- | --- |
|  |  | **Listen to/play music** | **Paint, draw, or make things** | **Read for enjoyment** | |
| **Most days vs otherwise** | **High parental engagement in Sweep 4** | | | |  |
|  | **ATT** | 0.087 (0.022)** | 0.133 (0.032)** | 0.159 (0.033)** | |
|  | **Mean bias %** | 0.8 | 0.7 | 1.0 | |
|  | **Rubin’s B** | 5.9 | 5.1 | 5.9 | |
|  | **Rubin’s R** | 1.17 | 1.01 | 1.13 | |
|  | **Treatment N** | 4830 | 1318 | 2312 | |
|  | **Control N** | 3028 | 3680 | 2199 | |
|  | **Total N** | 7858 | 4998 | 4511 | |
|  | **Low parental engagement in Sweep 4** | | | |  |
|  | **ATT** | 0.085 (0.058) | 0.096 (0.041)🕆 | 0.087 (0.054) | |
|  | **Mean bias (%)** | 1.2 | 0.8 | 1.2 | |
|  | **Rubin’s B** | 7.0 | 5.5 | 6.7 | |
|  | **Rubin’s R** | 1.07 | 1.16 | 1.08 | |
|  | **Treatment N** | 647 | 831 | 812 | |
|  | **Control N** | 604 | 3280 | 1213 | |
|  | **Total N** | 1251 | 4111 | 2025 | |
| **Most days vs never/less often than once a month** | **High parental engagement in Sweep 4** | | | |  |
|  | **ATT** | 0.216 (0.058)** | 0.328 (0.053)** | 0.338 (0.063)** | |
|  | **Mean bias (%)** | 2.4 | 2.6 | 2.8 | |
|  | **Rubin’s B** | 15.1 | 16.4 | 16.0 | |
|  | **Rubin’s R** | 1.29 | 1.45 | 1.43 | |
|  | **Treatment N** | 4830 | 1318 | 2312 | |
|  | **Control N** | 569 | 927 | 510 | |
|  | **Total N** | 5399 | 2245 | 2822 | |
|  | **Low parental engagement in Sweep 4** | | | |  |
|  | **ATT** | 0.178 (0.088)🕆 | 0.246 (0.053)** | 0.175 (0.068)🕆 | |
|  | **Mean bias (%)** | 3.6 | 2.1 | 3.1 | |
|  | **Rubin’s B** | 22.6 | 12.6 | 18.9 | |
|  | **Rubin’s R** | 1.24 | 1.13 | 1.33 | |
|  | **Treatment N** | 647 | 831 | 812 | |
|  | **Control N** | 177 | 982 | 379 | |
|  | **Total N** | 824 | 1813 | 1191 | |
| Notes: Columns present ATT estimates from PSM models using Epanechnikov kernel matching with 0.05 bandwidths; common support condition is imposed. The models controlled all covariates. ATT standard errors in parentheses were computed by bootstrapping with 100 replications.  Statistical significance is denoted by: 🕆 sig at 5%, ** sig at 0.1%.  Success of the propensity score matching was assessed using Rubin’s B<25%, Rubin’s R of 0.5-2, and a percentage bias of <10% for each covariate. | | | | | |
