# Supplementary material for: Arts engagement and self‐esteem in children: results from a propensity score matching analysis
Source: Ann N Y Acad Sci. 2019 Apr 15;1449(1):36–45. doi: 10.1111/nyas.14056 (PMC6767447; doi:10.1111/nyas.14056)
Supplement: Supplementary file 3 — Table S3. Relationship between ability rated by teacher (above average/well above average versus average or below) and self‐esteem in Sweep 5 among children who engage in arts and cultural activities most days (age 11) [file NYAS-1449-36-s003.docx]

| **Table S3 Relationship between ability rated by teacher (above average/well above average vs average or below) and self-esteem in Sweep 5 among children who engage in arts and cultural activities most days (age 11)** | | | |
| --- | --- | --- | --- |
| **Arts and cultural activities** | **Listen to/play music** | **Paint, draw, or make things** | **Read for enjoyment** |
| **Ability rated by teacher** | **Music** | **Arts & design** | **English language** |
| **ATT** | 0.111 (0.043)🕆 | -0.004 (0.061) | 0.128 (0.045)* |
| **Mean bias (%)** | 0.9 | 1.8 | 1.6 |
| **Rubin’s B** | 5.6 | 11.1 | 12.0 |
| **Rubin’s R** | 0.99 | 1.22 | 0.94 |
| **Treatment N** | 849 | 465 | 1554 |
| **Control N** | 2213 | 718 | 1035 |
| **Total N** | 3062 | 1183 | 2589 |
| Notes: Columns present ATT estimates from PSM models using Epanechnikov kernel matching with 0.05 bandwidths; common support condition is imposed. The models controlled all covariates. ATT standard errors in parentheses were computed by bootstrapping with 100 replications.  Statistical significance is denoted by: 🕆 sig at 5%, * sig at 1%  Success of the propensity score matching was assessed using Rubin’s B<25%, Rubin’s R of 0.5-2, and a percentage bias of <10% for each covariate. | | | |
